# Cystic renal oncocytoma: A rare case report

**DOI:** 10.1016/j.eucr.2021.101827

**Published:** 2021-08-27

**Authors:** Houcine Bouchaala, Mohamed Amine Mseddi, Mouna Zghal, Ibrahim Mejdoub, Lobna Ayedi, Mourad Hadj Slimen

**Affiliations:** a Urology Department, Academic Hospital Habib Bourguiba, Sfax, Tunisia; b Pathology Department, Academic Hospital Habib Bourguiba, Sfax, Tunisia

**Keywords:** Benign renal tumor, Cystic renal tumor, Oncocytoma, Bosniak classification

## Abstract

Cystic renal lesions are extremely common.

The major clinical concern is differentiating simple renal cysts from complex cysts to assess the risk of malignancy.

The Bosniak classification of renal cystic tumors is employed to distinguish benign cysts from potential malignant cysts.

Benign renal tumors can be rarely encountered in Bosniak type 4 cysts.

Herein, we report a case of 56-year-old female with a single right mediorenal solid-cystic mass classified bosniak 4. An open surgery was planned: There was a 2-cm-sized cystic tumor, mediorenal, in contact with the hilum. A lumpectomy was performed.

Anatomopathological examination revealed a cystic oncocytoma.

## 1 Introduction

Cystic renal tumors are a diagnostic challenge. Both benign and malignant tumors can present as renal cysts.

Complex renal cysts are considered malignant tumors. They are usually diagnosed as renal cell carcinoma with cystic changes. Rarely, benign tumors of the kidney can present as complex cyst.^1^

Oncocytoma is a benign epithelial renal tumor, which usually presents as a solid tumor with a central stellate scar. Cystic oncocytoma is rare and only few cases have been reported in literature.

We report a case of a renal oncocytoma which presented as a Bosniak 4 renal cyst. Postoperative histopathological examination revealed the final diagnosis.

## 2 Case presentation

A 56-year-old women with a history of postoperative pulmonary embolism admitted to our hospital because of a right renal mass incidentally detected by computed tomography (CT) during a general checkup. Plain abdominal CT showed single right mediorenal solid-cystic mass measuring 22*24 mm, round and well limited, with a thin and irregular wall, with at least one thick septum and some raised wall nodules.

**Fig. 1:**
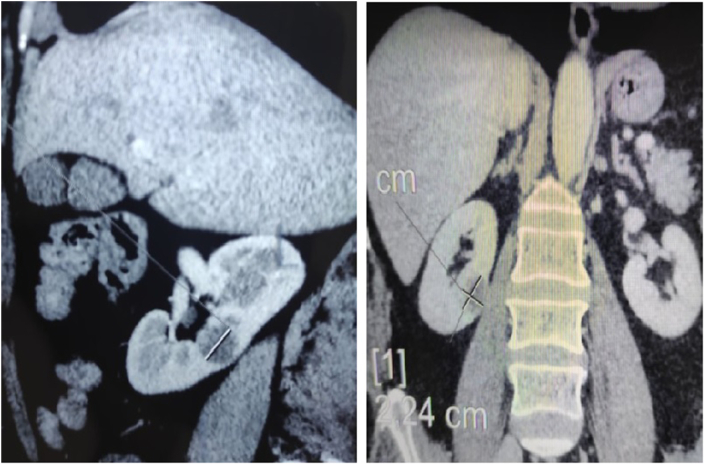
Plain abdominal computed tomography (CT) shows single right mediorenal solid-cystic mass measuring 22*24 mm, round and well limited.

The enhancement noted in the solid component was 94 Hounsfield Units and it was 9 Hounsfield Units in the cystic component. tumor classified bosniak 4 (Fig. 1).

There was no history of fever, hematuria, loss of weight or appetite. Abdominal examination was unremarkable. Her renal function tests, serum electrolytes, and hemoglobin and blood counts were within normal limits.

In view of the Bosniak type 4 cyst, an open lumpectomy was planned after obtaining written informed consent from the patient for partial nephrectomy as well as radical nephrectomy.

The right kidney was approached through the right flank eleventh transcostal lombotomy.

There was a 2-cm-sized gray-colored cystic tumor, mediorenal, in contact with the hilum. a lumpectomy was performed with macroscopically safe limit.

**Fig. 2:**
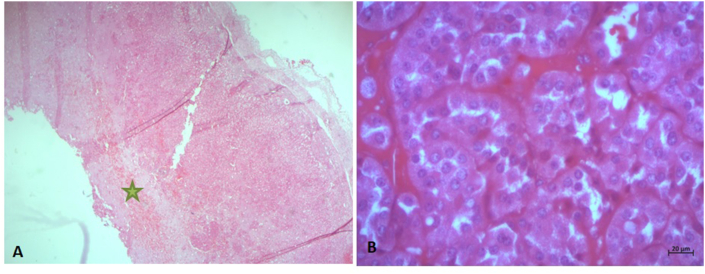
(A) Cystic wall formed by a tumor proliferation, centered by a hyalinizing fibrous scar( )(HE x 25), (B) Polygonal tumor cells with granular eosinophilic cytoplasm and regular round nucleoli (HEx 400).

Histological examination of the tumor showed a proliferation organized in nests or islets. the tumor cells are polygonal with abundant, eosinophilic, granular cytoplasm (Fig. 2). the nucleus is regular round and finely nucleated. the stroma is reduced.

**Fig. 3:**
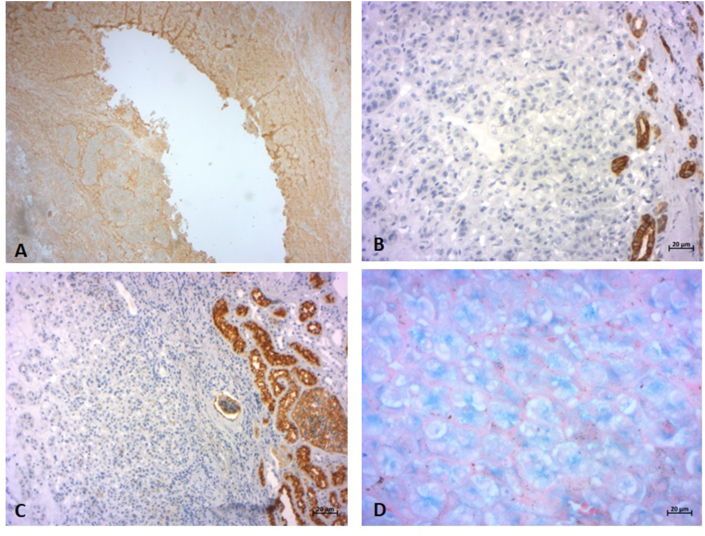
(A) Positive immunostaining for c-Kit (x 25) (B) Negative immunostaining for CK 7 (x200) (C) Negative immunostaining for CD 10 (x 100) (D) HALE stain: apical cytoplasmic positivity (x 400).

The tumor is centered by a hypocellular, hyaline fibrosis with some hemorrhagic suffusions. The tumor cells did not take up Hale's colloidal iron stain (Fig. 3).

Immunohistochemical staining was partially positive for C-Kit. it was negative for CD10, CK7 and E-Catherin confirming the diagnosis of an oncocytoma (Fig. 3).

## 3 Discussion

Renal oncocytoma is an uncommon benign tumor derived from the epithelial cells of the renal collecting tubules, commonly large with granular eosinophilic cytoplasm.

Oncocytoma accounts for approximately 5–7% of all solid renal tumors.^1^

The tumor can occur at any age. However, it is most common in the sixth decade. Men are more frequently affected with a sex ratio of 1,63 to 2,8.

Renal oncocytoma is rarely multifocal and exceptionally bilateral.

Morphological features of most renal tumors are well described and usually do not require ancillary tests. Nevertheless, renal oncocytoma may be challenging^2^

The main differential diagnosis is renal cell carcinoma, making its preoperative diagnosis rare.^1^^,^^3^

In some cases, the mass shows heterogeneous enhancement, suspect for clear cell renal cell carcinoma. It can also have a homogenous presentation just like papillary or chromophobe renal cell carcinoma.^4^

Consequently, these renal neoplasms are often treated operatively like renal cell carcinoma with radical or partial nephrectomy.

Renal oncocytoma usually presents as a well circumscribed mahogany-tan tumor. A central stellate scar with radiating fibrous septa is a common finding in oncocytoma, without evidence of hemorrhage or necrosis. However, atypical histological features have been described. Kodama et al. reported^2^ a case of renal oncocytoma with central cystic degeneration.

Furthermore, Skenderi et al.^3^ have reported twenty-four cases of cystic renal oncoytoma. As a microscopic cystic architecture may occur in oncocytoma, macrocystic renal oncocytoma is a rare entity.

Other atypical features have been reported in the literature, such as focal papillae, vascular invasion and perinephric fat invasion.

In a clinicoptahlogical study of renal oncocytoma reported by Omiyale et al.^5^ 159 cases of resected renal oncocytomas were included: 20 (12.6%) had vascular and/or perinephric fat invasion.

Immunohistochemical analysis of the tumor may be useful. Renal oncocytoma stains focally or negatively for cytokeratin-7 and negatively for vimentin and cytokeratin 20(CK 20).

## 4 Conclusion

Clinicians should be aware of the fact that Bosniak type 4 cysts can uncommonly be benign.

Most renal oncocytomas are usually diagnosed as malignant renal cell carcinoma by clinicians or radiologist.

Histological investigations are imperative in excluding malignancy in such conditions.

## Consent

Consent of the patient for publishing was obtained.

## Declaration of competing interest

No conflict of interest to be noted.
